# Supplementary figures and images for: Current and lagged associations of meteorological variables and Aedes mosquito indices with dengue incidence in the Philippines
Source: PLoS Negl Trop Dis. 2024 Jul 23;18(7):e0011603. doi: 10.1371/journal.pntd.0011603 (PMC11296630; doi:10.1371/journal.pntd.0011603)

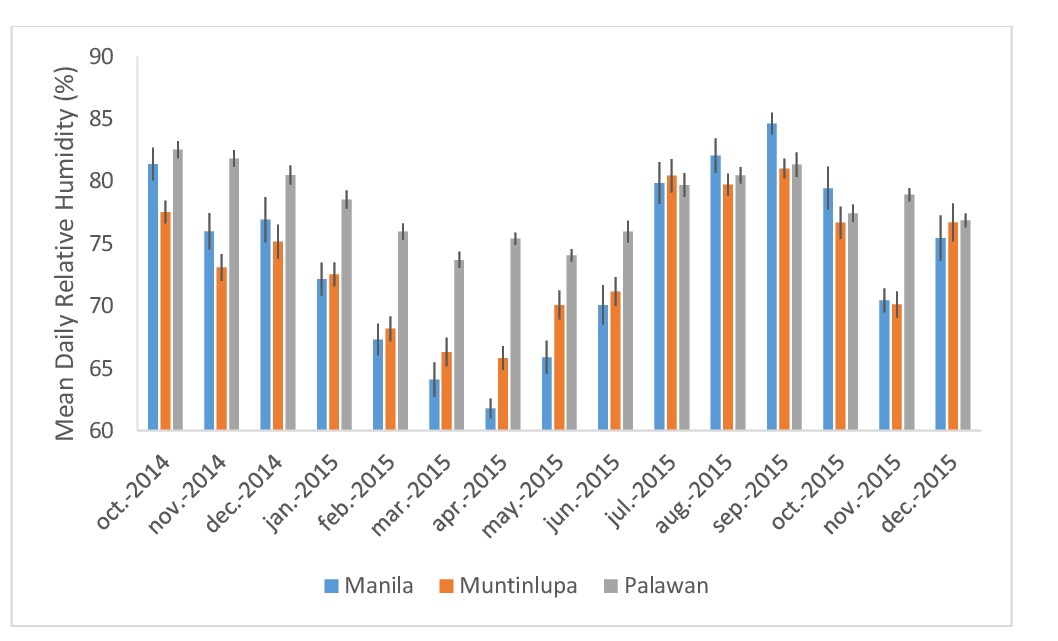

Supplement: S2 Fig — Shown are means and standard errors of the mean. (JPG) [file pntd.0011603.s002.jpg]

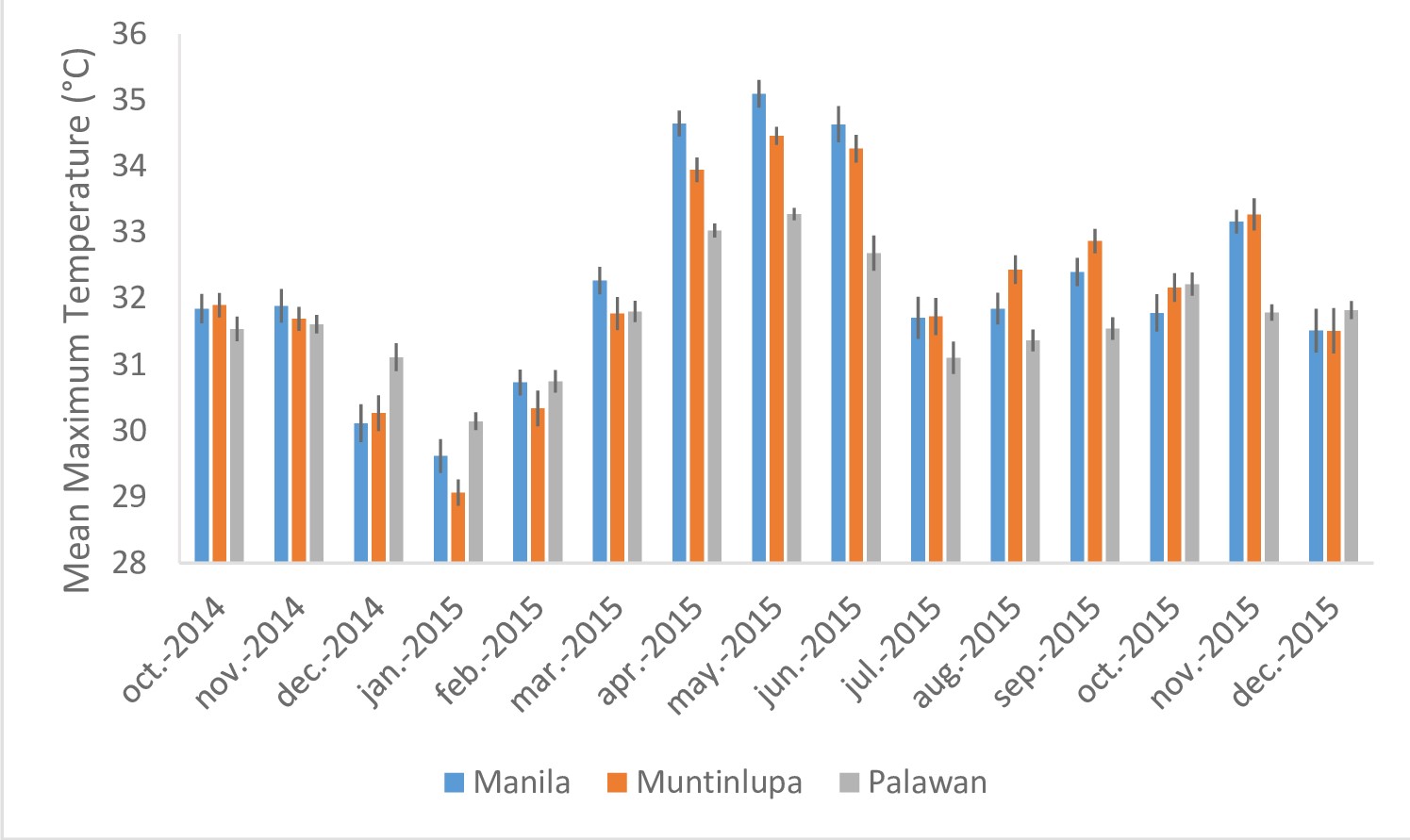

Supplement: S3 Fig — Shown are means and standard errors of the mean. (JPG) [file pntd.0011603.s003.jpg]

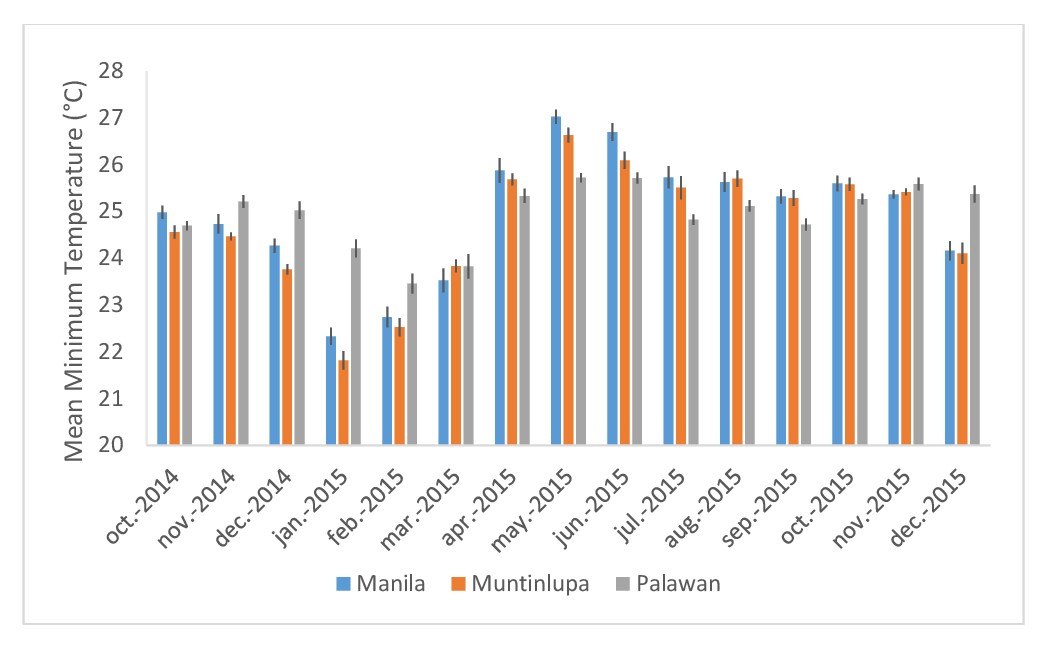

Supplement: S4 Fig — Shown are means and standard errors of the mean. (JPG) [file pntd.0011603.s004.jpg]

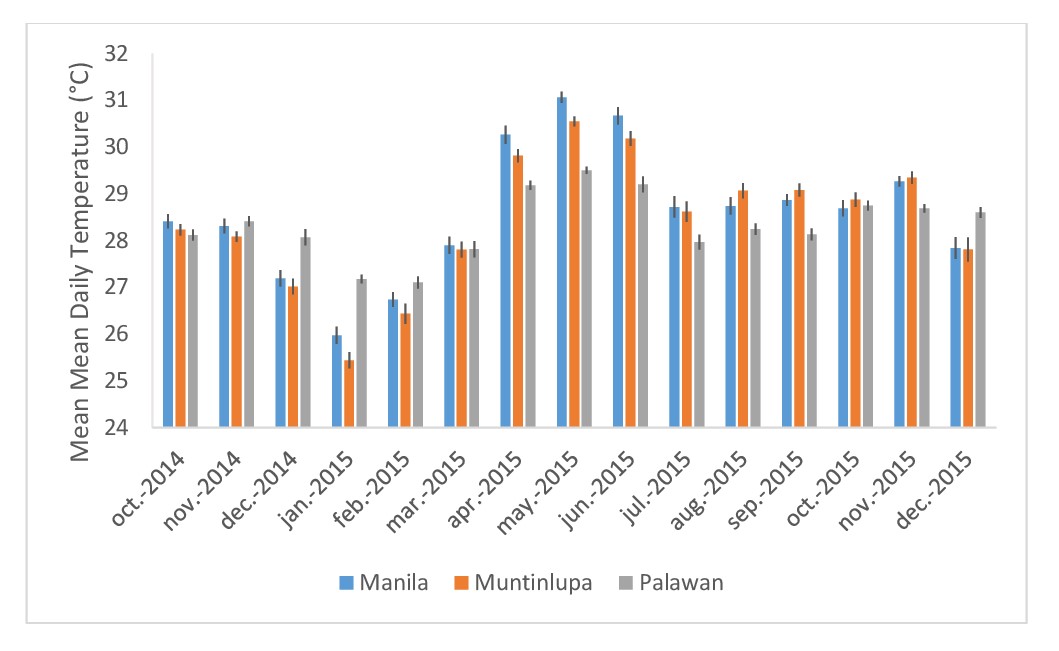

Supplement: S5 Fig — Shown are means and standard errors of the mean. (JPG) [file pntd.0011603.s005.jpg]

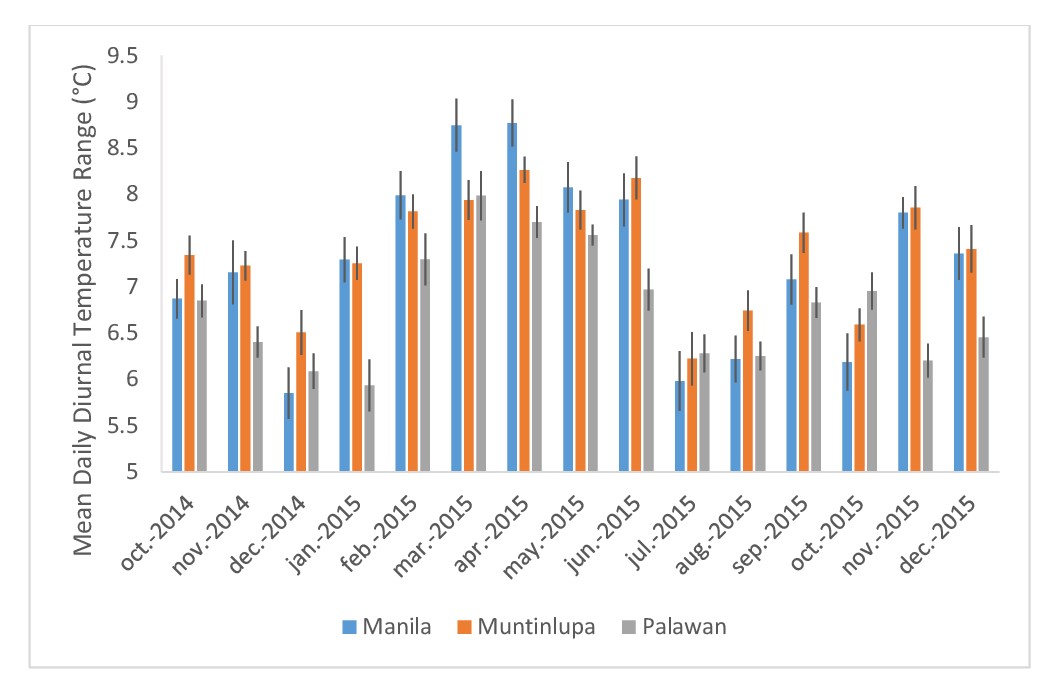

Supplement: S6 Fig — Shown are means and standard errors of the mean. (JPG) [file pntd.0011603.s006.jpg]

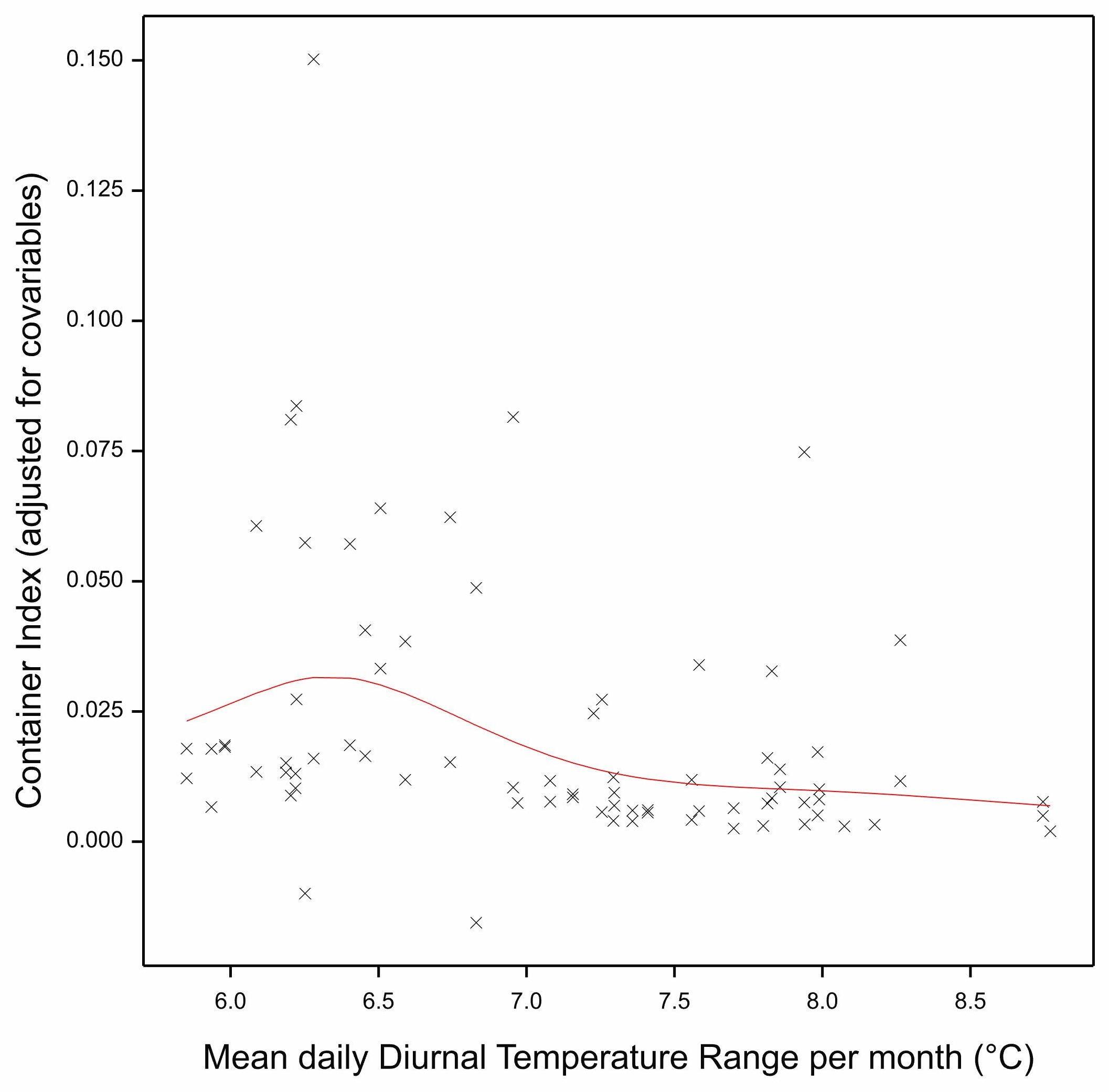

Supplement: S9 Fig — Red line shows the model output for the association of DTR with CI and the actual data are crosses. (JPG) [file pntd.0011603.s009.jpg]
