# Supplementary material for: Current and lagged associations of meteorological variables and Aedes mosquito indices with dengue incidence in the Philippines
Source: PLoS Negl Trop Dis. 2024 Jul 23;18(7):e0011603. doi: 10.1371/journal.pntd.0011603 (PMC11296630; doi:10.1371/journal.pntd.0011603)
Supplement: S1 Table — For each index we do 4 (lag weeks) x7 (meteorological variables) univariable analyses plus 1 multi per lag week and one combining all the lags together so 33. Bonferroni correction P value = 0.0015. P values in italics are those above this P value threshold. (DOCX) [file pntd.0011603.s014.docx]

|  | Variable | P value | variance explained (%) | | Sum % explained | |
| --- | --- | --- | --- | --- | --- | --- |
| House Index (Lag 1 wk) | Mean Rain (mm) | <.001 | 15.24 |  | | |
|  | Cumulative Rain (mm) | *0.039* | 3.74 | 18.98 | | |
| House Index (Lag 2 wks) | Mean DTR (°C) | <.001 | 18.26 |  | | |
|  | Cumulative Rain (mm) | 0.001 | 5.75 |  | | |
|  | Mean RH (%) | *0.004* | 4.92 |  | | |
|  | Max. Temp. (°C) | <.001 | 6.17 | 35.10 | | |
| House Index (Lag 3 wks) | Min. Temp. (°C) | *0.002* | 8.66 |  | | |
|  | Cumulative Rain (mm) | *0.013* | 5.85 | 14.52 | | |
| House Index (Lag 4 wks) | Max. Temp. (°C) | <.001 | 6.75 |  | | |
|  | Cumulative Rain (mm) | <.001 | 12.48 |  | | |
|  | Mean DTR (°C) | <.001 | 6.96 |  | | |
|  | Mean RH (%) | <.001 | 7.78 | 33.97 | | |
| House Index (Combined lags) | Cumulative Rain (mm) Lag wk 1 | *0.004* | 14.86 |  | | |
|  | Mean DTR (°C) lag wk 2 | *0.028* | 9.90 | 24.76 | | |
| Container Index (Lag 1 wk) | Cumulative Rain (mm) | <.001 | 8.63 |  | | |
|  | Mean DTR (°C) | *0.015* | 4.36 |  | | |
|  | Mean Rain (mm) | *0.003* | 5.98 |  | | |
|  | Mean Temp. (°C) | <.001 | 9.85 | 28.82 | | |
| Container Index (Lag 2 wks) | Cumulative Rain (mm) | <.001 | 10.03 |  | | |
|  | Mean DTR (°C) | <.001 | 10.20 |  | | |
|  | Mean Temp. (°C) | <.001 | 10.49 | 30.72 | | |
| Container Index (Lag 3 wks) | Cumulative Rain (mm) | *0.026* | 6.01 |  | | |
|  | Mean DTR (°C) | *0.028* | 5.92 |  | | |
|  | Mean RH (%) | *0.035* | 5.56 | 17.49 | | |
| Container Index (Lag 4 wks) | Cumulative Rain (mm) | <.001 | 9.43 |  | | |
|  | Mean DTR (°C) | <.001 | 9.68 |  | | |
|  | Mean Temp. (°C) | 0.001 | 7.50 |  | | |
|  | Min. Temp. (°C) | *0.013* | 3.92 | 30.53 | | |
| Container Index (Combined lags) | Cumulative Rain (mm) Lag wk 1 | <.001 | 8.63 |  | | |
|  | Mean DTR (°C) lag wk 1 | *0.006* | 4.36 |  | | |
|  | Mean Rain (mm) lag wk 1 | <.001 | 5.98 |  | | |
|  | Mean Temp. (°C) lag wk 1 | <.001 | 9.85 |  | | |
|  | Mean RH (%) lag wk 1 | *0.012* | 3.72 |  | | |
|  | Cumulative Rain (mm) Lag wk 2 | <.001 | 11.13 |  | | |
|  | Mean DTR (°C) lag wk 2 | *0.002* | 5.05 | 48.72 | | |
| Breteau Index (Lag 1 wk) | Cumulative Rain (mm) | <.001 | 9.73 |  | | |
|  | Mean DTR (°C) | 0.001 | 6.23 |  | | |
|  | Mean Rain (mm) | *0.004* | 5.21 |  | | |
|  | Mean RH (%) | <.001 | 8.61 |  | | |
|  | Min. Temp. (°C) | <.001 | 9.01 | 38.80 | | |
| Breteau Index (Lag 2 wks) | Cumulative Rain (mm) | <.001 | 9.41 |  | | |
|  | Mean DTR (°C) | <.001 | 12.41 |  | | |
|  | Mean Temp. (°C) | <.001 | 11.52 |  | | |
|  | Max. Temp. (°C) | *0.037* | 1.98 |  | | |
|  | Min. Temp. (°C) | 0.001 | 4.16 |  | | |
|  | Mean RH (%) | *0.003* | 4.56 | 44.03 | | |
| Breteau Index (Lag 3 wks) | Cumulative Rain (mm) | *0.006* | 6.77 |  | | |
|  | Mean DTR (°C) | <.001 | 9.40 |  | | |
|  | Mean Temp. (°C) | *0.007* | 6.64 |  | | |
|  | Mean RH (%) | *0.008* | 6.40 | 29.21 | | |
| Breteau Index (Lag 4 wks) | Cumulative Rain (mm) | <.001 | 12.06 |  | | |
|  | Mean DTR (°C) | <.001 | 9.65 |  | | |
|  | Mean Rain (mm) | *0.045* | 3.26 |  | | |
|  | Mean Temp. (°C) | <.001 | 10.42 |  | | |
|  | Min. Temp. (°C) | *0.007* | 4.12 | 39.52 | | |
| Breteau Index (Combined lags) | Cumulative Rain (mm) Lag wk 3 | <.001 | 6.77 |  | |  |
|  | Mean DTR (°C) lag wk 3 | <.001 | 9.40 |  | |  |
|  | Mean Temp. (°C) lag wk 3 | <.001 | 6.64 |  | |  |
|  | Mean RH (%) lag wk 3 | <.001 | 6.40 |  | |  |
|  | Cumulative Rain (mm) Lag wk 2 | <.001 | 8.02 |  | |  |
|  | Mean DTR (°C) lag wk 2 | <.001 | 5.76 |  | |  |
|  | Mean Temp. (°C) lag wk 2 | <.001 | 13.76 |  | |  |
|  | Min. Temp (°C) lag wk 2 | *0.026* | 2.25 | 59.00 | |  |
| Pupal Index (Lag 1 wk) | Cumulative Rain (mm) | *0.004* | 9.65 |  | | |
|  | Mean DTR (°C) | <.001 | 19.84 |  | | |
|  | Mean RH (%) | *0.006* | 8.91 | 38.41 | | |
| Pupal Index (Lag 2 wks) | Cumulative Rain (mm) | <.001 | 21.79 |  | | |
|  | Mean DTR (°C) | <.001 | 14.37 |  | | |
|  | Mean Rain (mm) | *0.025* | 4.85 |  | | |
|  | Mean Temp. (°C) | *0.003* | 7.39 |  | | |
|  | Mean RH (%) | *0.034* | 4.47 | 52.87 | | |
| Pupal Index (Lag 3 wks) | Cumulative Rain (mm) | <.001 | 26.45 |  | | |
|  | Mean DTR (°C) | <.001 | 8.81 |  | | |
|  | Mean Rain (mm) | <.001 | 10.23 |  | | |
|  | Mean Temp. (°C) | *0.006* | 6.08 | 51.58 | | |
| Pupal Index (Lag 4 wks) | Cumulative Rain (mm) | <.001 | 15.06 |  | | |
|  | Mean DTR (°C) | *0.02* | 8.07 | 23.14 | | |
| Pupal Index (Combined lags) | Cumulative Rain (mm) Lag wk 4 | <.001 | 15.06 |  | | |
|  | Mean DTR (°C) Lag wk 4 | *0.002* | 8.07 |  | | |
|  | Cumulative Rain (mm) Lag wk 3 | <.001 | 16.39 |  | | |
|  | Cumulative Rain (mm) Lag wk 2 | <.001 | 8.86 |  | | |
|  | Mean DTR (°C) Lag wk 3 | *0.011* | 5.64 | 54.03 | | |
| Pupa per Person (Lag 1 wk) | Cumulative Rain (mm) | *0.003* | 9.68 |  | | |
|  | Mean DTR (°C) | <.001 | 20.38 |  | | |
|  | Min. Temp. (°C) | *0.015* | 6.86 | 36.92 | | |
| Pupa per Person (Lag 2 wks) | Cumulative Rain (mm) | <.001 | 23.73 |  | | |
|  | Mean DTR (°C) | <.001 | 12.43 |  | | |
|  | Mean Rain (mm) | *0.022* | 4.81 |  | | |
|  | Max. Temp. (°C) | 0.003 | 7.39 | 48.37 | | |
| Pupa per Person (Lag 3 wks) | Cumulative Rain (mm) | <.001 | 25.04 |  | | |
|  | Mean DTR (°C) | <.001 | 9.20 |  | | |
|  | Mean Rain (mm) | <.001 | 9.20 |  | | |
|  | Mean Temp. (°C) | *0.006* | 6.17 | 49.61 | | |
| Pupa per Person (Lag 4 wks) | Cumulative Rain (mm) | <.001 | 16.07 |  | | |
|  | Mean DTR (°C) | *0.014* | 7.68 |  | | |
|  | Mean RH (%) | *0.039* | 5.95 | 29.70 | | |
| Pupal per Person (Combined lags) | Cumulative Rain (mm) Lag wk 4 | <.001 | 16.07 |  | | |
|  | Mean DTR (°C) Lag wk 4 | *0.002* | 7.68 |  | | |
|  | Mean RH (%) lag wk 4 | *0.009* | 5.95 |  | | |
|  | Cumulative Rain (mm) Lag wk 3 | <.001 | 10.77 |  | | |
|  | Cumulative Rain (mm) Lag wk 2 | <.001 | 8.81 |  | | |
|  | Mean DTR (°C) Lag wk 3 | *0.012* | 5.53 | 54.81 | | |
| Adult *Aedes* spp. (Lag 1 wk) | Cumulative Rain (mm) | <.001 | 8.60 |  | | |
|  | Mean DTR (°C) | <.001 | 36.43 |  | | |
|  | Mean Rain (mm) | *0.012* | 5.37 |  | | |
|  | Mean RH (%) | <.001 | 9.23 |  | | |
|  | Min. Temp. (°C) | *0.049* | 3.75 | 63.38 | | |
| Adult Aedes spp. (Lag 2 wks) | Cumulative Rain (mm) | <.001 | 28.86 |  | | |
|  | Max. Temp. (°C) | <.001 | 10.67 |  | | |
|  | Min. Temp. (°C) | *0.023* | 5.55 | 45.08 | | |
| Adult Aedes spp. (Lag 3 wks) | Cumulative Rain (mm) | *0.007* | 6.28 |  | | |
|  | Mean RH (%) | *0.003* | 7.38 |  | | |
|  | Min. Temp. (°C) | <.001 | 31.50 |  | | |
|  | Max. Temp. (°C) | <.001 | 11.40 | 56.56 | | |
| Adult Aedes spp. (Lag 4 wks) | Cumulative Rain (mm) | <.001 | 10.32 |  | | |
|  | Mean DTR (°C) | <.001 | 24.49 |  | | |
|  | Mean Temp. (°C) | <.001 | 12.46 |  | | |
|  | Min. Temp. (°C) | *0.009* | 4.01 |  | | |
|  | Mean RH (%) | <.001 | 12.20 | 63.49 | | |
| Adult Aedes spp. (Combined Lags) | Cumulative Rain (mm) Lag wk 4 | <.001 | 10.32 |  | | |
|  | Mean DTR (°C) Lag wk 4 | <.001 | 24.49 |  | | |
|  | Mean Temp. (°C) lag wk 4 | <.001 | 12.46 |  | | |
|  | Min. Temp (°C) lag wk 4 | *0.01* | 4.01 |  | | |
|  | Mean RH (%) lag wk 4 | <.001 | 12.20 |  | | |
|  | Cumulative Rain (mm) Lag wk 2 | *0.026* | 3.98 | 67.46 | | |
