# Supplementary material for: Current and lagged associations of meteorological variables and Aedes mosquito indices with dengue incidence in the Philippines
Source: PLoS Negl Trop Dis. 2024 Jul 23;18(7):e0011603. doi: 10.1371/journal.pntd.0011603 (PMC11296630; doi:10.1371/journal.pntd.0011603)
Supplement: Attachment — Submitted filename: PNTD_manuscript.docx [file pntd.0011603.s015.docx]

PNTD-D-23-01056

This is a nice and timely manuscript analyzing the relationships between weather conditions, entomological indices and dengue counts in Philippines.

The presentation is relevant and quite up to date. See suggestion to include new papers on the same topic below.

A few comments:

- Overall, the manuscript abstract, methods and results could be a little bit improved for clarity. For example, the results could be presented and interpreted consistently through the pathway of weather – entomology – dengue and weather -dengue explaining the relationships, the strengths of the links, and the lag relationships for each variable and configuration.
- It is not clear what model was used to pool the data from the different districts, and if random effects where used. Standard is two stage hierarchical approaches for this type of analyses. Overall, the models should be better described and the handling of data from different settings and pooling of results should be better explained.
- The table are not always easy to follow and the models could maybe be supplied
- The versions of abstracts presented doesn´t appear to be identical.
- Log-linear refers to Poisson with or without over-dispersion?
- Figures of explanatory variables and entomology and dengue as outcomes should be shown for different lags, perhaps overlayed as different curves for different lags. Currently entomology is missing.
- What is the ability of the models to predict beyond fitted data?
- Figure 6 and 7 seems not super illustrative, please add legends in the figure with the color explanation. Would be good to see if this remains on “testing data”. Maybe enough with one figure like this and overlaying?
- Interesting to note that the DTR effects seems rather strong on Breteau index.

While rain on the adults, but please clarify in the table 1 what lags are considered, and what the results correspond to in terms of model and explanatory variables.

- *“Whilst Container, House and Breteau indices and cumulative adult mosquitoes (over 2 months) were all associated with dengue cases in the same month in the univariable analyses, only the latter was positively associated with dengue cases in the final multivariable analysis”*

This is interesting, but what lags were used? And the interpretation should be consider from the perspective that the weather variables are causing parts of the entomological variation in time (as shown) and thus entomology is on the causal pathway of weather/climate. This could perhaps mean that the entomological factors doesn’t seem to add more to predicting dengue counts than the parts of entomology that can be explained by the weather variables. Therefore, when having both weather and entomology in the model for explaining dengue counts, the entomological variables doesn’t add much.

- The below studies look at the same issues but using more data and should be discussed in terms of findings and interpretations:
- Liyanage P, Tozan Y, Overgaard HJ, Tissera HA, Rocklöv J. Effect of El Niño–Southern Oscillation and local weather on Aedes dvector activity from 2010 to 2018 in Kalutara district, Sri Lanka: a two-stage hierarchical analysis. The Lancet Planetary Health. 2022 Jul 1;6(7):e577-85.
- Liyanage P, Tozan Y, Tissera HA, Overgaard HJ, Rocklöv J. Assessing the associations between Aedes larval indices and dengue risk in Kalutara district, Sri Lanka: a hierarchical time series analysis from 2010 to 2019. Parasites & Vectors. 2022 Aug 3;15(1):277.
