# Supplementary material for: Current and lagged associations of meteorological variables and Aedes mosquito indices with dengue incidence in the Philippines
Source: PLoS Negl Trop Dis. 2024 Jul 23;18(7):e0011603. doi: 10.1371/journal.pntd.0011603 (PMC11296630; doi:10.1371/journal.pntd.0011603)
Supplement: Attachment — Submitted filename: ReviewerComments_Cruzetal_Aedes.docx [file pntd.0011603.s016.docx]

**Reviewer comments on ‘Current and lagged associations of meteorological variables and Aedes mosquito indices with dengue incidence in the Philippines’**

**Overall description:**

This study investigates the impact of meteorological variables and mosquito indices on dengue incidence, to support the development of early warning and response systems. While the general findings are not particularly novel (as there are now countless studies investigating climate and weather impacts on mosquito-borne disease transmission), these relationships are likely place- and context-specific, thus conducting this investigation in key cities in the Philippines is of merit. Further, the authors found a strong relationship between recent weather conditions, mosquito abundances and dengue cases which could be useful for developing local early warning systems. However, it is very challenging to rigorously evaluate the specific findings presented here as there are many key methodological details and figures missing (outlined further below),

**Major comments:**

- I would like to evaluate whether the qualitative descriptions provided in the discussion match the data, however the relationships between climate variables and mosquito indices are not depicted anywhere (only the statistical significance is reported in the tables. Further, it is hard to follow the results given the high number of models and specifications used here. Namely, is there a separate model regressing each of the mosquito indices against meteorological variables (6 models + an additional 24 models for the 1-4 week lags). Within these 30 models, are there separate univariate and multivariate models? Is there then another 6 models (+ lagged versions) using each of these mosquito indices as the univariate predictor of dengue cases? And then one additional model using all meteorological variables and mosquito indices as predictors of dengue? The results are described succinctly, which is helpful, but it is difficult to understand which model each result is derived form.
- It is surprising that using 1-4-week lagged temperature variables explained less variation than the non-lagged temperature variables (as it is very often the case that temperature and rainfall in the *prior* month have a larger impact on mosquito abundance than the equivalent variables in the current month). More methodological details are critical for making sense of these results. Namely, are the dengue cases used here attributed to the month in which it was reported? If so, does it not likely reflect transmission that occurred in the weeks prior? Further, were the models re-fit using these lagged variables? Or were the same variables as identified originally used and then various lags applied? I think the former would be more appropriate, as you wouldn’t necessarily expect the same variable to be important across time scales (e.g., precipitation may matter more in the 3-4 weeks prior as it would impact breeding habitat, but max temperature may matter more in the 1-2 weeks prior). This also applies to the model regressing dengue cases against meteorological variables.
- I might expect slightly different meteorological variables to be important for *Aedes aegypti* vs *albopictus* (particularly as *albopictus* has lower heat tolerance). It would be helpful to know if models generated for *Ae. aegypti* matched those generated using the two species combined.
- It would be helpful to know if any environmental data was collected from inside the homes themselves. I understand it is not possible to back and collect this information (and it would no longer be relevant), but prior work has shown there can be substantial differences in temperature in indoor and weather-station recorded temperatures (e.g., Pena-Garcia et al. 2023)

**Minor comments:**

- Line 65 – citation needed for these case counts in the Philippines.
- line 66 – typo (extra word ‘incidence’)
- Line 75 – could also add Ryan et al. 2019 (Plos NTD)
- Line 80 – the overall impacts of temperature on mosquito life history traits is also nicely summarized in Mordecai et al. 2020 (Ecology Letters)
- Line 87 – suggest either defining / describing what Integrated Vector Management practices and Communication for Behavioral Impact approaches are, or removing these details
- Line 92 – perhaps worth noting that a description of these indices (‘House, Container, Breteau and Pupal’) can be found in the methods
- Line 145 – could you add further details about surveillance of these water-holding containers. Namely, how far from the house did your survey extend? What types of natural and artificial containers did you inspect?
- Throughout – suggest using consistent language of either ‘entomological indices’ or ‘mosquito indices’ (they appear to be used inter-changeably and I was confused whether they were the same thing)
- Line 167 – the container and house indices are described as counts in the prior methods section – are they indeed proportions? Please clarify.
- Statistical analysis – is it correct that separate models were fit using each of the aforementioned mosquito indices as the outcome? (e.g., 5 models, one for HI, CI, etc.). And then another 5 models using these mosquito indices as the univariate predictor of dengue cases? And then one additional model using all meteorological variables and mosquito indices as predictors of dengue. Please clarify.
- Line 226 – what is meant by the variance explained by location? Is this referring to the barangay covariate in the model?
- Figures – perhaps it is just my version, but the figures (particularly figure 1) are very low resolution, making them difficult to read
- Figure 2 - can you use similar colors for barangays from the same city (e.g., dark and light blue for Manila, dark and light green for Muntinlupa, etc.)
- Line 254 – The units of PI and PPI are different, correct? I’m confused about the percentage reported here. Which index does this specifically refer to? (I assume PI?)
- Line 267 – What does it mean that the relationship with the meteorological variables is simpler? Fewer variables were significant?
- Line 277 – the description of the lagged-meteorological variable models should be included in the methods, with further details discussed (e.g., were models re-fit using lagged variables? Or were the same variables as identified originally used, and then various lags applied?)
- Line 290 – same comment as above – it would be helpful to know how exactly these lagged models were fit.
- Figures 3-4 – it would be helpful to provide the regression equation on this plot
- Line 336 – how is relative risk being calculated here? Recommend including details of this in the methods (e.g., what is the ‘standardized’ mosquito number).
- Line 339 – it is quite surprising that there are *no* associations of mosquito indices with dengue cases. I assume this is because the meteorological variables are highly correlated with the mosquito indices (as described in the prior paragraphs), and take away explanatory power, when it is likely the case that the meteorological variables impact the juvenile and adult mosquito abundance, which in turn drives dengue cases, rather than independent effects of these predictors).
- Line 349 – It would be very helpful to show these results in a figure. For example, a multi-panel figure showing some of the most important non-linear relationships between meteorological variables and mosquito indices seems important (as only statistical significance is captured in tables 1 & 2).
- Line 353 – missing word
- Line 360 – it would be very interesting to parse apart the relationship between *natural* and *artificial* containers. Namely, other studies have found the number of artificial containers increase under drought conditions, leading to increased suitable habitat for the Ae. aegypti (which has a high affinity for artificial breeding sources). I’m not sure if this information was collected, or if all containers were considered equal.
- Line 363 – what does ‘impacted by’ mean here – can you explain more specifically how these traits typically vary with increasing DTR?
- Throughout – cumulative should be lower case
- Line 369 – typo
- Line 377 – typo
- Line 390 – beyond in which direction?
- Paragraph starting on 385 – Mordecai et al. 2019 seems like a key citation missing from this paragraph. Namely, this study compiled experimental data on mosquito life history trait performance at different constant temperatures to identify the thermal optima, maxima, and minima for transmission of a variety of mosquito-borne diseases. It

found the thermal optima for *Ae. aegpyti*-vectored dengue transmission occurs at 29.1 C (and thermal maxima and minima of 34.5 and 17.8, respectively). This seems in alignment with the results presented here (although the temperature variables considered are not entirely equivalent).

- Line 398 – it seems important to note here that it is lower DTR *in warm locations* that likely aids dengue transmission, as the impact of DTR depends on what the baseline temperature is. At cold baseline temperatures, larger daily fluctuations typically increase transmission, while at high baseline temperatures, the same fluctuations typically decrease inhibit transmission (due to the non-linear relationships, and in particular the typical left skew, between trait performance and temperature). This is somewhat addressed in the remainder of the paragraph, but clarification of this point in the first two sentences would help.
- Line 410 – typo
- Mosquito indices – it was mentioned that both 1 month and 2 month cumulative adult mosquito abundances were noted, but then it is unclear which results are being presented. For example, on line 411 – is this result reference to the 1 or 2 month adult abundance?
- Line 413 – typo
- Paragraph starting on line 375 – it also seems pertinent to mention that lagged meteorological variables show a strong correspondence with dengue cases in Palawan (as shown in Figure 6).
- Line 416 – I agree that it would be valuable to examine the impacts of meteorological variables on these two species separately – why was this not done?
- Line 421 – the time delays discussed here critically miss the delays in infection and reporting.
