# Supplementary material for: Current and lagged associations of meteorological variables and Aedes mosquito indices with dengue incidence in the Philippines
Source: PLoS Negl Trop Dis. 2024 Jul 23;18(7):e0011603. doi: 10.1371/journal.pntd.0011603 (PMC11296630; doi:10.1371/journal.pntd.0011603)
Supplement: Attachment — Submitted filename: Respones to reviewers.docx [file pntd.0011603.s017.docx]

Dear Editor,

Please find below an exhaustive series of responses to questions and points raised by the reviewers, which were overall very helpful in refining the manuscript, especially in the suggested additional literature to provide even more context for our results. We have substantially clarified the methods, both with respect to the mosquito indices and the statistical analyses, as well as providing much more detailed information on the numbers of different species of larval and adult mosquitoes collected in the differing sites. With regards to the request to show all graphs for all analyses, we have attempted to find a suitable balance between presenting key figures showing the associations of meteorological variables with mosquito indices and dengue cases (and between the two latter also), without providing an endless series of graphics that would be very repetitive, unhelpful to the reader and likely off-putting. Given the rather limited time series, we have down-played the forecasting angle, but do wish to maintain the conclusion that the mosquito indices bring little added value to associations with dengue cases beyond that achieved by the meteorological variables that are far easier to obtain. We have however added several new figures in the manuscript and run all the figures through PACE.

We hope our extensive revisions sufficiently address the points raised and look forward to hearing from you.

Sincerely

Richard Paul on behalf of the authors.

Responses in black to Questions in red

1. How close were the meteorological stations to the selected cities? And how likely are they to be representative of climatic conditions in the cities?

A matter of kilometres. However, the main point here is that we wished to see the extent to which easily available meteorological data could be used for early warning. We are well aware that the microclimate is much more important, but this sort of data are never going to be available without a significant investment and a better calibration of satellite data with the microclimate. We are attempting this, but will take time. We have discussed this to a certain extent in the limitations paragraph, but we have now also added this sentence:

“However, efforts to calibrate local microclimate with satellite data would be a major step in addressing the real associations of meteorological variables with mosquito indices.”

2. It seems interesting that the proportions of immature Aedes sp were very different to adult Aedes mosquitos in the study sites (85.4% vs 50.5% for Aedes aegypti), do the authors have any intuition of why that may be?

This is because of the differing oviposition site preferences of Aedes vs. Culex spp. The immature mosquito indices are specifically designed to track Aedes spp. and not Culex spp. Adult mosquitoes of either genera will, however, have similar host questing behavior for a blood meal. We have added this sentence after mention of these percentages in the Results.

“The differences in the relative percentages of Culex and Aedes spp. for their immature and adult stages reflects the different oviposition site preferences of these genera and the fact that the mosquito immature indices are specifically designed for Aedes spp.”

3. It would be useful to understand how dengue incidence in the study year (2014-2015) compared to other dengue seasons - would it be considered an outbreak year? Similarly, it would be useful to understand the climate context of the study year chosen - were there any impacts from the 2014-2016 El Niño event?

2014 and 2015 were on the low side of reported cases (3800 and 13000 cases).

https://www.statista.com/statistics/1120319/philippines-number-dengue-cases/

There was an El Niño event in 2015-2016.

We have added this information in the Methods section.

“It subsequently transpired that the number of dengue cases during these two years were lower than average [10], despite there being an El Niño event during 2015-2016.”

4. As authors comment in the manuscript, this is a short time series to characterise associations between meteorological variables and dengue, it would be useful to see a sensitivity analysis around the analyses focusing on climate and dengue with a longer time series (as I assume the entomological data is only available from 2014-2015)?

The real purpose of the paper was to assess the added value of the mosquito indices. This is not really a climate dengue paper per se.

5. I am curious as to why authors decided to use loglinear regression rather than Poisson or negative binomial regression for analyses concerning counts? Similarly, the arcsine transformation is not commonly used and it would be useful to understand why this was chosen.

We have clarified this in the text. A GAM with a spline function to capture non-linearities and a Poisson log-linear regression was used. We use the broader definition of McCullagh and Nelder (1989, pp 193-194) take a broad view of the log-linear model, which they define as: log (mu_i) = eta_i = beta^T x_i	i = 1, ..., n	(eq 6.2) where beta^T is beta transpose. They say, "All log-linear models have the form (6.2). Variety is created by different forms of model matrices; there is an obvious analogy with analysis-of-variance and linear regression models." McCullagh, P and Nelder JA (1989) Generalized Linear Models. 2nd Edition. Chapman and Hall, London. Pp526

For the arc sine query: We have to transform the mosquito indices data when used as an explanatory variable because they are not normally distributed. The arc sine transformation is one way to do this for percentage/ proportion data.

6. In the methods section authors describe how the multivariable model was selected through a backwards selection process until a final adequate model with only significant variables was achieved. I’m interested as to whether other model adequacy criteria were used in model selection to avoid overfitting, e.g. WAIC? It would also be useful to understand whether this process was repeated for every outcome assessed.

We appreciate the overfitting issue but when fitting a GAM with a spline function and implementing an overdispersion factor, as the model is not estimated by maximum likehood we can’t actually compute an AIC. We have tried to clarify the model fitting procedure in the methods in more detail.

7. In the methods section authors describe how highly correlated and colinear variables were excluded but final models seem to include several highly related variables (e.g. many models contain both cumulative rain and mean rain, while the model for Pupal index contains both minimum and maximum temperature). This would suggest that some of the final estimates of association between meteorological variables and the outcome may be confounded, could the authors add some more information on which colinear and correlated variables were excluded and which were kept in?

No variables were ever excluded based on collinearity. Rather we retained all those that remained significant even if they are related. We also re-fitted models where collinearity was suspected, replacing such variables one by one to assess the best variable. It is certainly true that for example minimum and maximum temperature might well be having the same biological effect. However, again the main object of the paper was to assess the added value of mosquito indices for dengue alert.

8. The methods section also describes that “barangay was nested within city”, was this through a random effect? Model equations for the GAMs used (even if in the supplementary) would be very helpful in understanding the model structure.

No, City+ City.Barangay were fitted as fixed effects as we wished to see how they differed. Barangay was only thus fitted as an interaction term with City.

We could add an example model in the supplementary material. Such as for dengue cases:

Cases~Poisson (μt)

log(μt) = α + β1(City)t + β2(City.Barangay)t + s(Maximum Temperature, λ)t

where log(μt) is the logarithm of expected dengue cases at time point t, α is the model intercept, β are the beta coefficients and s(variable, λ) is the natural cubic spline smoothing function

9. I assume that model results shown (tables of % variance explained and figures) were from the final multivariate model for each outcome but this is unclear and it would be helpful to add this information into the table and figure legends.

This has been added.

10. With regards to lagged meteorological variables, from Table 2 it seems that each meteorological variable was assessed at the same lag. This does not seem ideal as it’s likely that different meteorological variables will affect dengue incidence at differing time lags. It would be useful to see results from models tested with meteorological variables at different lags, particularly if an aim of this paper is to examine potentially useful models for early warning.

The lags were combined but proved even less explanatory than the individual lags, themselves worse than the current month’s meteorological associations. However, for sake of completion we will add this information.

We have added the following in the text and added the individual variable information to Table S1: “When performing a combined analysis of all the significant variables identified in the individual four week lag times did not improve the model fit (% variance explained Current month vs lagged fits: HI: 57% vs. 25%; CI: 51% vs. 40%: BI: 63% vs.57%; PI: 49% vs. 40%; PPI: 49% vs. 36%; Adults: 72% vs.60%).”

--------------------

11. Reviewer #2: The results are described succinctly, but there are no corresponding figures, thus is it not possible for me to evaluate whether the descriptions match the data. The figures are provided at very low resolution.

We are bit confused here as we have 19 Supplementary Figures and 7 figures in the text. We will attempt to improve the resolution of the Figures generally through using the PLoS PACE.

12. Reviewer #3: 11. With regards to the figures showing associations between meteorological variables and mosquito / dengue outcomes, it would be preferable to show the non-linear association on the relative risk scale. This would help the viewer to interpret the magnitude of the non-linear effect of each meteorological variable on mosquito abundance / dengue risk.

We beg to differ. One of the agreeable features of GAMs is displaying the non-linearity and highlighting the key values at which any non-linearity occurs. This is particularly the case here for the DTR, where peak incidence values, for example, can clearly be seen. However, whilst the RR would be of interest for dengue cases, we feel that to show the actual relationship between the variables is more understandable, particularly regarding the non-linear relationship. Moreover, given the somewhat limited time series, it is probably pushing things a bit far to start assigning RR to significantly associated variables. The relationships identified are interesting and do support previous studies to some extent. The concluding point that mosquito indices do not bring added value to dengue prediction is one of the aims to test in this study and despite the limitations they don’t seem to bring anything in the way of added explanatory power.

13. It is somewhat confusing to only see the results for some of the explanatory variables in the figures - it would be preferable to see all the results for one model as a figure, with panels for each association between the explanatory variable and the outcome, with similar figures for other outcomes in the Supplementary. This would help the reader interpret the relative effects of each variable on the outcome. It would also be useful to show confidence intervals in the results figures.

There are a lot of figures in both the text and the supplementary material and so we wished to emphasize those associations that were most interesting. There are so many models that if we put in all the figures it would seriously detract from the main messages. However, we have added several new Figures concerning the most pertinent variables.

14. Figures 6 and 7 are somewhat confusing for the reader, it would be preferable to show each site as a separate line or as panels of the Figure rather than alternating between sites on the x-axes.

This has been done.

--------------------

15. Reviewer #3: In Line 442 authors conclude that “the link between mosquito indices and dengue cases is non-significant”. It would be useful to see the model results for models with mosquito indices and dengue cases but without meteorological variables. From the current results, it seems this paper is showing that they don’t provide any additional explanation of variance when added to a meteorological model, which would be expected under a causal pathway in which meteorological variables affect dengue incidence through their effect on the vector population. It would be useful to add some discussion on the causal hypotheses examined here. Given that the study design in this paper is based on explaining variation in dengue incidence rather than predicting cases, it is also possible that the inclusion of mosquito indices could still be useful in a predictive early warning model and I would recommend amending this conclusion.

There is a section, starting on line 331 giving the association results of mosquito variables and dengue cases. However, we agree that the discussion should be amended as suggested.

The last sentence has been cut and replaced with “This absence of added value of mosquito indices is likely because of the effects of the meteorological variables on the mosquito bionomics. It is possible, however, that at finer spatial scales mosquito indices may bring additional predictive value, being affected by the microclimate that the meteorological stations cannot capture. Further studies focussing on the more local scale with respect to both microclimate and mosquito indices might be a promising route of investigation.”

--------------------

16. Reviewer #3: I think there was an issue with rendering in Figure 1 making the text difficult to read, some of the text on map inserts is illegible.

Figure 1 has been re-done to improve clarity.

--------------------

17.	I would like to evaluate whether the qualitative descriptions provided in the discussion match the data, however the relationships between climate variables and mosquito indices are not depicted anywhere (only the statistical significance is reported in the tables).

There are quite a few graphs, mostly in the supplementary material. We wished to avoid presenting too many graphs from all the models carried out. However, we appreciate that some more may be useful and have thus inserted graphs on the key associations. The larval/pupal indices (HI, CI, BI) all showed the same pattern, which we state in the text and just show the relationships for HI (and one supplementary one for CI).

18. Further, it is hard to follow the results given the high number of models and specifications used here. Namely, is there a separate model regressing each of the mosquito indices against meteorological variables (6 models + an additional 24 models for the 1-4 week lags). Within these 30 models, are there separate univariate and multivariate models? Is there then another 6 models (+ lagged versions) using each of these mosquito indices as the univariate predictor of dengue cases? And then one additional model using all meteorological variables and mosquito indices as predictors of dengue? The results are described succinctly, which is helpful, but it is difficult to understand which model each result is derived form.

For the association of meteorological variables with each mosquito index: there are 7 meteorological variables, thus giving 7 univariable and 1 multivariable analyses per index (thus 8). There are 5 “lag” time points (0, 1, 2, 3 4 wks), thus 8x5=40 plus a combined analysis including all the 4 lags (lag wks 1-4). This is indicated in the Table footnote and have now also added this in the methods.

For associations with dengue, we approached this differently because the time scale for a putative effect of meteorological or entomological variables on dengue cases was deemed to be longer. For this reason we assessed the association of the variables on current month’s dengue cases and those occurring one and two months later. This has been explained more fully in the methods.

19. It is surprising that using 1-4-week lagged temperature variables explained less variation than the non-lagged temperature variables (as it is very often the case that temperature and rainfall in the prior month have a larger impact on mosquito abundance than the equivalent variables in the current month). More methodological details are critical for making sense of these results. Namely, are the dengue cases used here attributed to the month in which it was reported? If so, does it not likely reflect transmission that occurred in the weeks prior?

For the associations of meteorological variables with mosquito indices, with the exception of the two pupal indices where the lags did have more explanatory power, we agree that this the weekly lags explained less well the mosquito indices. We have discussed this (in the discussion).

For dengue cases, a different approach was used as mentioned above.

20. Further, were the models re-fit using these lagged variables? Or were the same variables as identified originally used and then various lags applied? I think the former would be more appropriate, as you wouldn’t necessarily expect the same variable to be important across time scales (e.g., precipitation may matter more in the 3-4 weeks prior as it would impact breeding habitat, but max temperature may matter more in the 1-2 weeks prior). This also applies to the model regressing dengue cases against meteorological variables.

The models were indeed re-fit using these lagged variables. We have attempted to make this clearer in the methods and we have included the combined lag analyses of all the meteorological variables on mosquito indices and added this information in the results section and Table S1.

21. I might expect slightly different meteorological variables to be important for Aedes aegypti vs albopictus (particularly as albopictus has lower heat tolerance). It would be helpful to know if models generated for Ae. aegypti matched those generated using the two species combined.

This was done but not explicitly so. *Ae. albopictus* was only present in the Palawan sites where it was predominant. As outlined from Line 267, the meteorological associations carried out only in Palawan were very similar to those found previously. There were only 16 occasions when albopictus was found alone (in the absence of aegypti) in any container, thereby not offering much in the way of comparison with aegypti and too few adults to do any useful analyses. This is now mentioned in the text (beginning of results section) and also discussed later.

22. It would be helpful to know if any environmental data was collected from inside the homes themselves. I understand it is not possible to back and collect this information (and it would no longer be relevant), but prior work has shown there can be substantial differences in temperature in indoor and weather-station recorded temperatures (e.g., Pena-Garcia et al. 2023)

This is a very valid point and we are well aware of the limitations of using meteorological station data as compared to the microclimate data. This has been emphasized in the limitations paragraph.

Minor comments:

23. Line 65 – citation needed for these case counts in the Philippines.

Modified and added reference.

24. line 66 – typo (extra word ‘incidence’)

Edited

25. Line 75 – could also add Ryan et al. 2019 (Plos NTD)

Added

26. Line 80 – the overall impacts of temperature on mosquito life history traits is also nicely summarized in Mordecai et al. 2020 (Ecology Letters)

Added

27. Line 87 – suggest either defining / describing what Integrated Vector Management practices and Communication for Behavioral Impact approaches are, or removing these details

Removed

28. Line 92 – perhaps worth noting that a description of these indices (‘House, Container, Breteau and Pupal’) can be found in the methods

Have indicated this in the text

29. Line 145 – could you add further details about surveillance of these water-holding containers. Namely, how far from the house did your survey extend? What types of natural and artificial containers did you inspect?

We have included the following: “Inspection was carried out within and immediately surrounding the house; i.e. in their yards/gardens. Containers inspected are majoritarily artificial, but can include organic waste (discarded coconut shells) and bamboo stumps of palm plant axials where present.”

30. Throughout – suggest using consistent language of either ‘entomological indices’ or ‘mosquito indices’ (they appear to be used inter-changeably and I was confused whether they were the same thing)

Changed to mosquito indices throughout

31. Line 167 – the container and house indices are described as counts in the prior methods section – are they indeed proportions? Please clarify.

They are percentages /proportions. This has been corrected in the text.

32. Statistical analysis – is it correct that separate models were fit using each of the aforementioned mosquito indices as the outcome? (e.g., 5 models, one for HI, CI, etc.). And then another 5 models using these mosquito indices as the univariate predictor of dengue cases? And then one additional model using all meteorological variables and mosquito indices as predictors of dengue. Please clarify.

We have clarified the analysis procedure in the stats section.

But yes, each mosquito index and each meteorological variable first analysed separately and then jointly in a multivariable model.

33. Line 226 – what is meant by the variance explained by location? Is this referring to the barangay covariate in the model?

City and the interaction term of City with Barangay. We have added this in parentheses after location.

34. Figures – perhaps it is just my version, but the figures (particularly figure 1) are very low resolution, making them difficult to read

Figure 1 has been re-done and the others checked for clarity using the PACE system of PLoS.

35. Figure 2 - can you use similar colors for barangays from the same city (e.g., dark and light blue for Manila, dark and light green for Muntinlupa, etc.)

Good suggestion. Changed.

36. Line 254 – The units of PI and PPI are different, correct? I’m confused about the percentage reported here. Which index does this specifically refer to? (I assume PI?)

Yes you are correct. PI is % and PPI is unitless. This has been corrected in the text.

37. Line 267 – What does it mean that the relationship with the meteorological variables is simpler? Fewer variables were significant?

Have edited this out

38. Line 277 – the description of the lagged-meteorological variable models should be included in the methods, with further details discussed (e.g., were models re-fit using lagged variables? Or were the same variables as identified originally used, and then various lags applied?)

This has been explained in detail in the methods

39. Line 290 – same comment as above – it would be helpful to know how exactly these lagged models were fit.

As above

40. Figures 3-4 – it would be helpful to provide the regression equation on this plot

These plots, as with all the plots showing associations are from the univariable analysis. Plots from the multivariable analyses are too complicated to understand. We have given in the methods the Poisson loglinear model used throughout.

41. Line 336 – how is relative risk being calculated here? Recommend including details of this in the methods (e.g., what is the ‘standardized’ mosquito number).

Details added to the methods.

Relative Risk from the Poisson is the exponentiated value of the model parameter estimates

Because the distribution of the data are not normal, the data were transformed either by using an arcsine transformation for the proportion/percentage indices or by standardization for the count indices (standardization is akin to normalization and is calculated as the subtraction of the mean from each data point and division by the standard deviation)

42. Line 339 – it is quite surprising that there are no associations of mosquito indices with dengue cases. I assume this is because the meteorological variables are highly correlated with the mosquito indices (as described in the prior paragraphs), and take away explanatory power, when it is likely the case that the meteorological variables impact the juvenile and adult mosquito abundance, which in turn drives dengue cases, rather than independent effects of these predictors).

Yes, that what we think is happening and thus mosquito indices do not bring any added value.

43. Line 349 – It would be very helpful to show these results in a figure. For example, a multi-panel figure showing some of the most important non-linear relationships between meteorological variables and mosquito indices seems important (as only statistical significance is captured in tables 1 & 2).

New Figures have been added to present the key variables.

44. Line 353 – missing word

“increasing values of indices”

45. Line 360 – it would be very interesting to parse apart the relationship between natural and artificial containers. Namely, other studies have found the number of artificial containers increase under drought conditions, leading to increased suitable habitat for the Ae. aegypti (which has a high affinity for artificial breeding sources). I’m not sure if this information was collected, or if all containers were considered equal.

There were so few natural containers in our setting as to make any robust analysis meaningful. However, the idea is very interesting.

46. Line 363 – what does ‘impacted by’ mean here – can you explain more specifically how these traits typically vary with increasing DTR?

We mean negatively affected. We have modified this to say:

Whilst larval and pupal developmental rates do increase with temperature, they have been shown to decrease with increasing DTR.

47. Throughout – cumulative should be lower case

OK

48. Line 369 – typo

Done

49. Line 377 – typo

We can’t find that typo

50. Line 390 – beyond in which direction?

Edited to “above”

51. Paragraph starting on 385 – Mordecai et al. 2019 seems like a key citation missing from this paragraph. Namely, this study compiled experimental data on mosquito life history trait performance at different constant temperatures to identify the thermal optima, maxima, and minima for transmission of a variety of mosquito-borne diseases. It

found the thermal optima for Ae. aegpyti-vectored dengue transmission occurs at 29.1 C (and thermal maxima and minima of 34.5 and 17.8, respectively). This seems in alignment with the results presented here (although the temperature variables considered are not entirely equivalent).

Thank you. Have inserted this reference and information.

52. Line 398 – it seems important to note here that it is lower DTR in warm locations that likely aids dengue transmission, as the impact of DTR depends on what the baseline temperature is. At cold baseline temperatures, larger daily fluctuations typically increase transmission, while at high baseline temperatures, the same fluctuations typically decrease inhibit transmission (due to the non-linear relationships, and in particular the typical left skew, between trait performance and temperature). This is somewhat addressed in the remainder of the paragraph, but clarification of this point in the first two sentences would help.

Have added this: “It should, however, be noted that increasing DTR has been predicted to increase vectorial capacity at lower temperatures (~14°C), highlighting the complex temperature effects on viral transmission [23].”

53. Line 410 – typo

Edited out the second “rain”

54. Mosquito indices – it was mentioned that both 1 month and 2 month cumulative adult mosquito abundances were noted, but then it is unclear which results are being presented. For example, on line 411 – is this result reference to the 1 or 2 month adult abundance?

Cumulative (2 monthly) adult mosquitoes were only used in the analysis of mosquito variables on dengue. For meteorological variables on mosquitoes, only that month’s adult mosquitoes were analysed. We have clarified this in the text.

55. Line 413 – typo

Don’t see the typo here

56. Paragraph starting on line 375 – it also seems pertinent to mention that lagged meteorological variables show a strong correspondence with dengue cases in Palawan (as shown in Figure 6).

We have added this: “Moreover, when we analysed the two islands separately, the lagged meteorological associations gave as good as, or even a better fit than the variables from the current month.”

57. Line 416 – I agree that it would be valuable to examine the impacts of meteorological variables on these two species separately – why was this not done?

We only had albopictus from Palawan and for that reason we did do some analyses separately for Palawan. There were however too few *albopictus* larvae/pupae occurring in the absence of *Ae. aegypti* to make any substantial difference in the mosquito indices even within the Palawan sites to be able to run a within-site comparative analysis with aegypti vs albopictus. We have added additional information in the text on this.

58. Line 421 – the time delays discussed here critically miss the delays in infection and reporting.

Have added in the reporting delays. The infection part is generally covered.

59. Overall, the manuscript abstract, methods and results could be a little bit improved for clarity. For example, the results could be presented and interpreted consistently through the pathway of weather – entomology – dengue and weather -dengue explaining the relationships, the strengths of the links, and the lag relationships for each variable and configuration.

We have attempted to improve clarity throughout in line with the revewiers’ suggestions.

60. It is not clear what model was used to pool the data from the different districts, and if random effects where used. Standard is two stage hierarchical approaches for this type of analyses. Overall, the models should be better described and the handling of data from different settings and pooling of results should be better explained.

We have better described the modelling approach and included a model example in the methods. We didn’t fit random effects as we were interested in the differences among islands and sites, especially given the occurrence of albopictus only in Palawan.

61. The table are not always easy to follow and the models could maybe be supplied

We have attempted to clarify the tables and have given a model as an example.

62. The versions of abstracts presented doesn´t appear to be identical.

This has been rectified

63. Log-linear refers to Poisson with or without over-dispersion?

With over-dispersion

64. Figures of explanatory variables and entomology and dengue as outcomes should be shown for different lags, perhaps overlayed as different curves for different lags. Currently entomology is missing.

We have added new figures of entomology on dengue.

65. What is the ability of the models to predict beyond fitted data?

We have not attempted prediction, because the time series is too short to then test our prediction. We have therefore removed he emphasis on prediction and replaced with association.

66. Figure 6 and 7 seems not super illustrative, please add legends in the figure with the color explanation. Would be good to see if this remains on “testing data”. Maybe enough with one figure like this and overlaying?

As asked by another reviewer we have separated the figures into those for each site. We do not attempt to split the data into model fitting and then prediction for testing, the time series being too short.

67. Interesting to note that the DTR effects seems rather strong on Breteau index.

While rain on the adults, but please clarify in the table 1 what lags are considered, and what the results correspond to in terms of model and explanatory variables.

Table 1 has no lags. This is now explained in the legend. The lag data table is Table S1.

68. *“Whilst Container, House and Breteau indices and cumulative adult mosquitoes (over 2 months) were all associated with dengue cases in the same month in the univariable analyses, only the latter was positively associated with dengue cases in the final multivariable analysis”*

This is interesting, but what lags were used? And the interpretation should be consider from the perspective that the weather variables are causing parts of the entomological variation in time (as shown) and thus entomology is on the causal pathway of weather/climate. This could perhaps mean that the entomological factors doesn’t seem to add more to predicting dengue counts than the parts of entomology that can be explained by the weather variables. Therefore, when having both weather and entomology in the model for explaining dengue counts, the entomological variables doesn’t add much.

The lags have been thoroughly described in the methods, and yes we agree that the absence of added value is because the meteorological variables are explaining the mosquito indices. We have stated this as clearly as possible in the discussion because it is a main part of the conclusions. Using easily available meteorological data seems to be a better use of resources than laboriously collecting mosquito data.

69. The below studies look at the same issues but using more data and should be discussed in terms of findings and interpretations:

Thank you for these refs. We have already included a similar work by Liyanage et al. 2016, but agree these papers are very pertinent and have been included and discussed.

Liyanage P, et al. The Lancet Planetary Health. 2022 Jul 1;6(7):e577-85.

Liyanage P, et al. Parasites & Vectors. 2022 Aug 3;15(1):277.
