# Supplementary material for: Current and lagged associations of meteorological variables and Aedes mosquito indices with dengue incidence in the Philippines
Source: PLoS Negl Trop Dis. 2024 Jul 23;18(7):e0011603. doi: 10.1371/journal.pntd.0011603 (PMC11296630; doi:10.1371/journal.pntd.0011603)
Supplement: Attachment — Submitted filename: ReviewerComments_Cruzetal_Aedes_Round2.docx [file pntd.0011603.s018.docx]

Round 2 responses and additional comments

- Original comment: 18. Further, it is hard to follow the results given the high number of models and specifications used here. Namely, is there a separate model regressing each of the mosquito indices against meteorological variables (6 models + an additional 24 models for the 1-4 week lags). Within these 30 models, are there separate univariate and multivariate models? Is there then another 6 models (+ lagged versions) using each of these mosquito indices as the univariate predictor of dengue cases? And then one additional model using all meteorological variables and mosquito indices as predictors of dengue? The results are described succinctly, which is helpful, but it is difficult to understand which model each result is derived form.
- Author responses: For the association of meteorological variables with each mosquito index: there are 7 meteorological variables, thus giving 7 univariable and 1 multivariable analyses per index (thus 8). There are 5 “lag” time points (0, 1, 2, 3 4 wks), thus 8x5=40 plus a combined analysis including all the 4 lags (lag wks 1- 4). This is indicated in the Table footnote and have now also added this in the methods. For associations with dengue, we approached this differently because the time scale for a putative effect of meteorological or entomological variables on dengue cases was deemed to be longer. For this reason we assessed the association of the variables on current month’s dengue cases and those occurring one and two months later. This has been explained more fully in the methods.
- Round 2 comment: I appreciate that the authors have attempted to clarify this in the methods. Is it not 8 models *per* index though, for a total of 40? Then, for each of these models, there are 5 lag time points for a total of 200?
- Original comment: 19. It is surprising that using 1-4-week lagged temperature variables explained less variation than the non-lagged temperature variables (as it is very often the case that temperature and rainfall in the prior month have a larger impact on mosquito abundance than the equivalent variables in the current month). More methodological details are critical for making sense of these results. Namely, are the dengue cases used here attributed to the month in which it was reported? If so, does it not likely reflect transmission that occurred in the weeks prior?
- Author responses: For the associations of meteorological variables with mosquito indices, with the exception of the two pupal indices where the lags did have more explanatory power, we agree that this the weekly lags explained less well the mosquito indices. We have discussed this (in the discussion).
  For dengue cases, a different approach was used as mentioned above.
- Round 2 comment: I appreciate that the authors have added information in the discussion regarding the relationship between lagged vs current weather variables and mosquito indices. However, I see no corresponding discussion concerning dengue *cases.* I remain confused as to whether dengue cases are ascribed to the month they were reported (thus inherently capturing a lag from transmission), or if some correction was applied. This should be discussed in the discussion and methods. I see the authors state on line 208 “For associations with dengue, we approached this differently because the time scale for a putative effect of meteorological or entomological variables on dengue cases was deemed to be longer.” Further details and references are needed here (i.e. the selection of 1-2 months, the presence of other climate-sensitive processes involved in transmission).
- Original comment: 45. Line 360 – it would be very interesting to parse apart the relationship between natural and artificial containers. Namely, other studies have found the number of artificial containers increase under drought conditions, leading to increased suitable habitat for the Ae. aegypti (which has a high affinity for artificial breeding sources). I’m not sure if this information was collected, or if all containers were considered equal.
- Author response: There were so few natural containers in our setting as to make any robust analysis meaningful. However, the idea is very interesting.
- Round 2 comment: Was data on the container type (or at least artificial vs natural) collected? If so, it would seem helpful, interesting, and relevant to include that in the manuscript.

Additional major comments:

- The authors state that this is not a climate-dengue paper, but rather an attempt to identify an early warning system for dengue based on climate. As such, the framing of the discussion seems off to me. There is a lot of emphasis on the relationship between specific climate variables and mosquito indices (*i.e.,* which temperature/precipitation variables are important, whether they are non-linear, and the extent to which they are lagged). However, including mosquito indices did not improve dengue case prediction. Therefore, I would think the abstract, introduction, and discussion of the manuscript should focus much more heavily on the factors predicting dengue cases. Along these lines, explaining that mosquito indices do not provide additional explanatory power could be a major point. That is, efforts to collect these indices may be unnecessary, and vector control resources should be applied elsewhere.
- The figures remain very low resolution in the available pdf, but perhaps this is only an issue for me.
