# Supplementary material for: Current and lagged associations of meteorological variables and Aedes mosquito indices with dengue incidence in the Philippines
Source: PLoS Negl Trop Dis. 2024 Jul 23;18(7):e0011603. doi: 10.1371/journal.pntd.0011603 (PMC11296630; doi:10.1371/journal.pntd.0011603)
Supplement: Attachment — Submitted filename: responses to ReviewerComments_Cruzetal_Aedes_Round2.docx [file pntd.0011603.s019.docx]

Round 2 responses and additional comments

Our responses to round 1 are in italics. Our responses to round 2 review are in normal font following the round 2 comments in red.

- Original comment: 18. Further, it is hard to follow the results given the high number of models and specifications used here. Namely, is there a separate model regressing each of the mosquito indices against meteorological variables (6 models + an additional 24 models for the 1-4 week lags). Within these 30 models, are there separate univariate and multivariate models? Is there then another 6 models (+ lagged versions) using each of these mosquito indices as the univariate predictor of dengue cases? And then one additional model using all meteorological variables and mosquito indices as predictors of dengue? The results are described succinctly, which is helpful, but it is difficult to understand which model each result is derived form.
- *Author responses: For the association of meteorological variables with each mosquito index: there are 7 meteorological variables, thus giving 7 univariable and 1 multivariable analyses per index (thus 8). There are 5 “lag” time points (0, 1, 2, 3 4 wks), thus 8x5=40 plus a combined analysis including all the 4 lags (lag wks 1- 4). This is indicated in the Table footnote and have now also added this in the methods. For associations with dengue, we approached this differently because the time scale for a putative effect of meteorological or entomological variables on dengue cases was deemed to be longer. For this reason we assessed the association of the variables on current month’s dengue cases and those occurring one and two months later. This has been explained more fully in the methods.*
- Round 2 comment: I appreciate that the authors have attempted to clarify this in the methods. Is it not 8 models *per* index though, for a total of 40? Then, for each of these models, there are 5 lag time points for a total of 200?

Response: For each mosquito index there are indeed 8 models per time point and as there are 5 time points (lags) there are 40 models per index. Then there is an additional multivariable analysis including all the lags together, thus giving 41 models per index. There are several indices, but as these outcome data are different, there is no need to implement a Bonferroni correction of the P value using the entire number of models performed across all the indices. We’re not sure how to make this clearer. However, we did notice that there was a 0 missing from the corrected P value in the methods, although the correct value is given in the footnote of the Table, which is likely the source of the confusion.

Thus, we have edited as follows “Therefore, overall, there were five “lag” time points (0, 1, 2, 3, 4 weeks), eight models per lag plus a combined analysis including all the four lags (lag wks 1-4) giving 41 models per mosquito index. The significance threshold P value of 0.05 was thus divided by 41, giving a Bonferroni P threshold of 0.0012.”

- Original comment: 19. It is surprising that using 1-4-week lagged temperature variables explained less variation than the non-lagged temperature variables (as it is very often the case that temperature and rainfall in the prior month have a larger impact on mosquito abundance than the equivalent variables in the current month). More methodological details are critical for making sense of these results. Namely, are the dengue cases used here attributed to the month in which it was reported? If so, does it not likely reflect transmission that occurred in the weeks prior?
- *Author responses: For the associations of meteorological variables with mosquito indices, with the exception of the two pupal indices where the lags did have more explanatory power, we agree that this the weekly lags explained less well the mosquito indices. We have discussed this (in the discussion).
  For dengue cases, a different approach was used as mentioned above.*
- Round 2 comment: I appreciate that the authors have added information in the discussion regarding the relationship between lagged vs current weather variables and mosquito indices. However, I see no corresponding discussion concerning dengue *cases.* I remain confused as to whether dengue cases are ascribed to the month they were reported (thus inherently capturing a lag from transmission), or if some correction was applied. This should be discussed in the discussion and methods. I see the authors state on line 208 “For associations with dengue, we approached this differently because the time scale for a putative effect of meteorological or entomological variables on dengue cases was deemed to be longer.” Further details and references are needed here (i.e. the selection of 1-2 months, the presence of other climate-sensitive processes involved in transmission).

Response: We have added the following to explain why the dengue cases were analyzed in the same month and one and two months later. There are many potential delays between the effects of the meteorological and entomological variables and the dengue cases:

“For associations with dengue, we approached this differently because the time scale for a putative effect of meteorological or entomological variables on dengue cases was deemed to be longer for several reasons. Firstly, the expansion of the mosquito population because of meteorological effects will take time and thus any consequent effect on virus transmission will be accordingly delayed. Secondly, because of the extrinsic incubation period (EIP). The first blood meal is generally taken three days after emergence of the adult mosquito [21]. Therefore, assuming an EIP of 7–12 days, a minimum of 10–15 days would be required for a newly emerged mosquito to become infectious if its first blood meal was on an infected person [39]. This additional delay will thus generate a lag between the mosquito indices and the dengue incidence. Thirdly, the probability of a mosquito having an infected bloodmeal will be low at the start of the epidemic and increase with increasing dengue incidences. Thus, there will be some delay during the initial phases of the epidemic prior to the expansion of the viral population within the community.”

- Original comment: 45. Line 360 – it would be very interesting to parse apart the relationship between natural and artificial containers. Namely, other studies have found the number of artificial containers increase under drought conditions, leading to increased suitable habitat for the Ae. aegypti (which has a high affinity for artificial breeding sources). I’m not sure if this information was collected, or if all containers were considered equal.
- *Author response: There were so few natural containers in our setting as to make any robust analysis meaningful. However, the idea is very interesting.*
- Round 2 comment: Was data on the container type (or at least artificial vs natural) collected? If so, it would seem helpful, interesting, and relevant to include that in the manuscript.

Response: We have added the following in the results:

“The most productive containers for immature stages in Manila and Muntinlupa were jugs/pitchers, dish drains, gallons, pails and drums. In Palawan, in addition to these container types, other productive containers were tyres, coconut shells, wells and garbage cans.”

Additional major comments:

- The authors state that this is not a climate-dengue paper, but rather an attempt to identify an early warning system for dengue based on climate. As such, the framing of the discussion seems off to me. There is a lot of emphasis on the relationship between specific climate variables and mosquito indices (*i.e.,* which temperature/precipitation variables are important, whether they are non-linear, and the extent to which they are lagged). However, including mosquito indices did not improve dengue case prediction. Therefore, I would think the abstract, introduction, and discussion of the manuscript should focus much more heavily on the factors predicting dengue cases. Along these lines, explaining that mosquito indices do not provide additional explanatory power could be a major point. That is, efforts to collect these indices may be unnecessary, and vector control resources should be applied elsewhere.

Response: The ultimate aim of identifying variables useful for EWS has to go through the procedure of testing the associations of locally measured meteorological (and mosquito) variables with dengue. In this sense there is an underlying notion of climate but analysed at a much more local scale than ENSO for example or other more large scale climate phenomena. Thus, at the end of the introduction we state our aims that cover the broad relationships among the different variables/indices: “The aim of this study was to expand the current knowledge on the associations between different meteorological variables and both the Aedes indices and the incidence of dengue and to assess the extent to which the mosquito indices, which are laborious to perform, bring added value to meteorological variables for explaining variation in dengue incidences in three representative cities in the Philippines.”

Indeed, one of the main purposes of the paper was to assess the added value of measuring the mosquito indices for any EWS. We have edited throughout to emphasise this more strongly, as indicated below. However, we do feel it necessary to include in the discussion the details on the shape and size of the relationships between meteorological variables, mosquito indices and dengue incidences to put our study into context and highlight what variables are associated and thus be useful for use in a EWS. Such detail also contributes to the body of work focusing on the relationships, providing some biological explanation and notably the novel inclusion of the DTR as well as adult mosquitoes, which are not traditionally collected measurements.

In the abstract we did actually say that mosquito indices are not useful and that two meteorological variables hold promise. We have edited as “ DTR and minimum temperature also explained variation in dengue cases occurring one and two months later and may offer potentially useful variables for an EWS.”

We have also attempted to emphasis throughout the focus on EWS.

In the discussion we have added the following to make the connection with EWS more evident.

“This study identified two meteorological variables, DTR and minimum temperature, which were found to be associated with dengue cases one and two months later. Previous analyses found, amongst other meteorological variables, mean or minimum temperature to be associated with dengue cases occurring one month, 2-3 months and 1-12 weeks later according to the study [35, 36, 57]. This consistent association of temperature being associated with increasing dengue incidences in geographically different settings (Thailand, Brazil, Mexico, Barbados) is very promising for its use in a EWS [35, 36, 57]. The clear associations of meteorological variables and the mosquito indices is encouraging and yet it seems that the link between such indices and dengue incidence remains difficult to detect. Only adult mosquitoes showed any positive association with dengue incidence, but far weaker than when using meteorological variables. Previous work examining the association between mosquito indices and dengue cases did reveal associations with BI, CI and HI at lags of 1-2 months, but did not consider simultaneously the effect of the meteorological variables [58]. Despite recommendations from stakeholders testing the most elaborate EWS to date, EWARS, to include mosquito indices, our study suggests that the indices bring no added value [35].”

And altered the final concluding paragraph as follows:

“In conclusion, there have been increasing efforts to establish early warning and response systems using meteorological information, but which although promising have often proven country-specific [34, 35, 57]. Our study does, however, suggest that there are globally consistent meteorological variables providing some predictive power at lead times of 1-2 months. It also reaffirms the lack of explanatory power of mosquito indices and hence questions the utility of investing the substantial effort necessary for collecting such indices. The absence of added value of mosquito indices may be because the meteorological variables strongly influence the mosquito bionomics and vectorial capacity and thereby better capture the associations with dengue incidence than mosquito indices can. It is possible, however, that at finer spatial scales mosquito indices may bring additional predictive value, being affected by the microclimate that the meteorological stations cannot capture. Further studies focussing on the more local scale with respect to both microclimate and mosquito indices might be a promising route of investigation.”

- The figures remain very low resolution in the available pdf, but perhaps this is only an issue for me.

Response: We’re not too sure what to do about the figures. We used the PLoS image system to get certified image quality. Not sure what else we can do.
